# Investigation of TLR2 and TLR4 Polymorphisms and Sepsis Susceptibility: Computational and Experimental Approaches

**DOI:** 10.3390/ijms231810982

**Published:** 2022-09-19

**Authors:** Mohammed Y. Behairy, Ali A. Abdelrahman, Eman A. Toraih, Emad El-Deen A. Ibrahim, Marwa M. Azab, Anwar A. Sayed, Hany R. Hashem

**Affiliations:** 1 Department of Microbiology and Immunology, Faculty of Pharmacy, University of Sadat City, Sadat City 32958, Egypt; 2 Department of Microbiology and Immunology, Faculty of Pharmacy, Suez Canal University, Ismailia 41522, Egypt; 3 Department of Surgery, School of Medicine, Tulane University, New Orleans, LA 70112, USA; 4 Genetics Unit, Department of Histology & Cell Biology, Faculty of Medicine, Suez Canal University, Ismailia 41522, Egypt; 5 Department of Anesthesia, Intensive Care and Pain Management, Faculty of Medicine, Suez Canal University, Ismailia 41522, Egypt; 6 Department of Medical Microbiology and Immunology, Taibah University, Madinah 42353, Saudi Arabia; 7 Department of Surgery and Cancer, Imperial College London, London SW7 2BX, UK; 8 Department of Microbiology and Immunology, Faculty of Pharmacy, Fayoum University, Fayoum 63514, Egypt

**Keywords:** TLR, polymorphism, infection, sepsis, septic shock

## Abstract

Toll-like receptors (TLR) play an eminent role in the regulation of immune responses to invading pathogens during sepsis. TLR genetic variants might influence individual susceptibility to developing sepsis. The current study aimed to investigate the association of genetic polymorphisms of the *TLR2* and *TLR4* with the risk of developing sepsis with both a pilot study and in silico tools. Different in silico tools were used to predict the impact of our SNPs on protein structure, stability, and function. Furthermore, in our prospective study, all patients matching the inclusion criteria in the intensive care units (ICU) were included and followed up, and DNA samples were genotyped using real-time polymerase chain reaction (RT-PCR) technology. There was a significant association between *TLR2* Arg753Gln polymorphisms and sepsis under the over-dominant model (*p* = 0.043). In contrast, we did not find a significant difference with the *TLR4* Asp299Gly polymorphism with sepsis. However, there was a significant association between *TLR4* Asp299Gly polymorphisms and *Acinetobacter baumannii* infection which is quite a virulent organism in ICU (*p* = 0.001) and post-surgical cohorts (*p* = 0.033). Our results conclude that the *TLR2* genotype may be a risk factor for sepsis in adult patients.

## 1. Introduction

Infection is one of the prominent causes of human morbidity and mortality, especially in patients requiring critical care [1,2]. Moreover, in intensive care units (ICUs), a serious complication of infection is sepsis and its maximal manifestation, septic shock [3]. Sepsis is an infection-induced life-threatening organ dysfunction with mortality rates reaching 20–70% [4,5].

Infectious diseases have been found to be a major selective pressure [6]. Despite the ambiguity of the precise etiology of sepsis, numerous studies have shown that gene polymorphisms have an important role in affecting individual susceptibility to sepsis [7]. Some polymorphisms of the innate immune system are supposed to mediate a predisposition to infectious complications including the outcome of patients with sepsis [8]. The innate immune system is of crucial importance for both the direct defense against micro-organisms and the activation of the adaptive immune system [9]. The innate immunity system is the main mediator of inflammation, and it recruits specific pattern recognition receptors (PRRs) capable of recognizing micro-organisms through identifying conserved pathogen-associated molecular patterns.

Toll-like receptors (TLR) are the most studied subtypes of pattern recognition receptors with their critical importance in the immune system [10,11]. Among the members of the TLR family, TLR2 and TLR4 are considered the most important PRRs that cover a wide range of antigenic determinants [12]. TLR4 has a distinctive ability to recognize a very wide range of microorganisms including Gram-negative bacteria through Lipopolysaccharide (LPS), in addition to many viruses and Fungi. Meanwhile, TLR2 is regarded as a key molecule in regulating our immune system with a crucial role in the recognition of Peptidoglycans of Gram-positive bacteria, in addition to different ligands of yeast, fungi, viruses, and parasites [12,13].

One of the most studied innate immunity polymorphisms is the *TLR4* Asp299Gly (rs4986790) polymorphism, which interferes with TLR4 signal transduction; thus, it is supposed to affect host susceptibility to infections and microbial invasions [14]. Moreover, structural analysis of *TLR4* Asp299Gly has revealed evidence of a resulted impairment in TLR4 binding to its ligands [15]. Meanwhile, one of the most important polymorphisms of *TLR2* is Arg753Gln; the presence of this SNP was found to impair the signaling pathway of this key receptor [16], thus suggesting increased susceptibility to infections and sepsis.

Consequently, many studies have been conducted all over the world to reveal the prevalence of these SNPs and their impact on infection and sepsis susceptibility, but a varied pattern of prevalence was found for both *TLR4* and *TLR2* SNPs among different populations [13,17,18]. In addition, conflicting results were found regarding their impact on infection and sepsis susceptibility in different populations [19,20,21]. Therefore, a need was felt for further investigation on these issues. The usage of computational approaches in studying SNPs’ impact has gained momentum and importance in recent years [22,23,24,25]. Integrating the in silico approach with the experimental one provides great accuracy and depth to the analysis.

In this study, we aimed to investigate the possible role of *TLR2* and *TLR4* polymorphisms in affecting sepsis susceptibility and survival in critically ill patients in the Egyptian population using both in silico analysis and experimental methods.

## 2. Results

**Figure 1:**
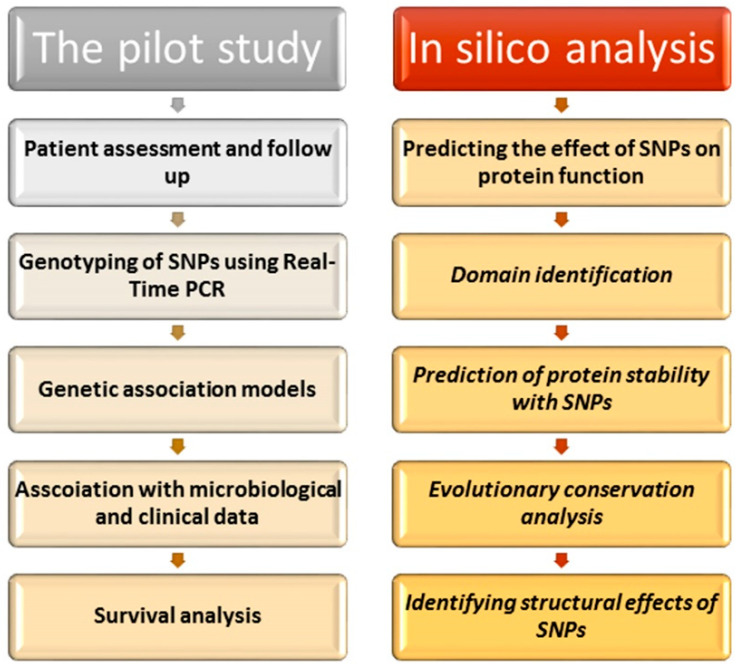
Scheme illustrating the outline of the study plan.

The study involved both a pilot study and in silico analysis. A scheme illustrating the layout of the study plan is shown in Figure 1.

### 2.1. In Silico Analysis

#### 2.1.1. General Information: TLR2

**Figure 2:**
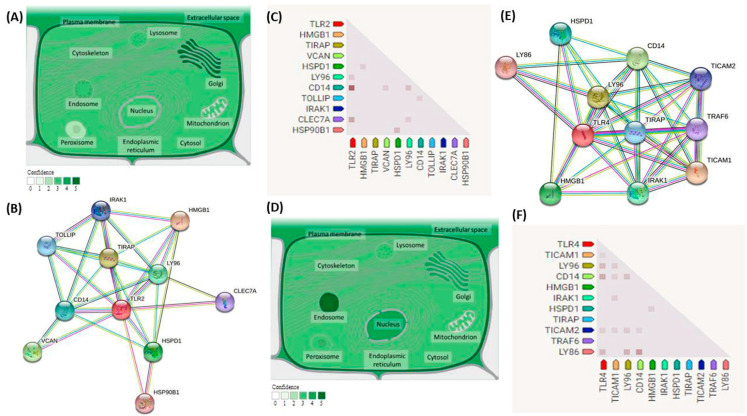
Functional Analysis of TLR2 and TLR4 proteins. (**A**) Subcellular localization of the TLR2 protein. The gradient of green color indicates the degree of confidence (genecards.org) with (compartments.jensenlab.org) as the source of the image. (**B**) Predicted network of protein–protein interactions of the TLR2 protein. Proteins are represented by nodes, while predicted associations are represented by edges that could be drawn with 7 colored lines that indicate different types of evidence. Redline fusion evidence, Light blue line—database evidence. Green line—neighborhood evidence. Blue line—co-occurrence evidence. Purple line—experimental evidence. Black line—coexpression evidence. Yellow line—text mining evidence. HMGB1: High mobility group protein B1, TIRAP: Toll/interleukin-1 receptor domain-containing adapter protein, VCAN: Versican core protein, HSPD1: 60 kDa heat shock protein, LY96: Lymphocyte antigen 96, CD14: Monocyte differentiation antigen CD14, TOLLIP: Toll-interacting protein, IRAK1: Interleukin-1 receptor-associated kinase 1, CLEC7A: C-type lectin domain family 7 member A, HSP90B1: Endoplasmin. STRING analysis (version 11.5). (**C**) *TLR2* Gene Coexpression matrix. Predict association between protein functions, Color intensity shows the confidence level in the association between protein functions. TLR2 shows coexpression with CD14, CLEC7A, and LY96 with scores of 0.611, 0.281, and 0.130, respectively (https://string-db.org (accessed on 29 August 2021)). (**D**) Subcellular localization of TLR4 protein (genecards.org) with (compartments.jensenlab.org) as the source of the image. (compartments.jensenlab.org). (**E**) 1B predicted a network of protein–protein interactions of the TLR4 protein. TICAM1: TIR domain-containing adapter molecule 1, TICAM2: TIR domain-containing adapter molecule 2, TRAF6: TNF receptor-associated factor 6, LY86: Lymphocyte antigen 86. STRING analysis (version 11.5). (**F**) *TLR4* Gene Coexpression matrix. TLR4 shows coexpression with LY86, CD14, and LY96 with scores of 0.301, 0.264, and 0.176, respectively (https://string-db.org (accessed on 29 August 2021)).

*TLR2* gene (ENSG00000137462) is a protein-coding gene located on 4q31.3. It is composed of five exons with a length of 26,564 nucleotides. It is located on Chromosome 4: 153684080-153710643 according to the Genome Reference Consortium Human Build 38 patch release 13 (GRCh38.p13) with NCBI Reference Sequence (NC_000004.12) (https://www.ncbi.nlm.nih.gov/gene/7097 (accessed on 29 August 2021)). There are eight transcripts for this gene (ensemble.org). This gene encodes Toll-like receptor 2 protein, a member of the Toll-like receptor (TLR) family. Figure 2A shows the subcellular localization of TLR2. The predicted network of protein–protein interactions of the TLR2 protein is shown in Figure 2B and its Gene Coexpression matrix (Figure 2C) shows coexpression with CD14, CLEC7A, and LY96 with scores of 0.611, 0.281, and 0.130, respectively (https://string-db.org (accessed on 29 August 2021)). Rs5743708 is an SNP located at Chromosome 4, position: 153705165 (forward strand) with two alleles (G and A). G is the ancestral allele and the minor allele frequency for A equals 0.01. This is a missense variant that causes the replacement of amino acid Arginine with amino acid Glutamine at position 753.

#### 2.1.2. General Information: TLR4

*TLR4* gene (ENSG00000136869) is a protein-coding gene located on 9q33.1. It is composed of four exons with a length of 20,333 nucleotides. It is located on Chromosome 9: 117704403-117724735 according to the Genome Reference Consortium Human Build 38 patch release 13 (GRCh38.p13) with NCBI Reference Sequence (NC_000009.12) (https://www.ncbi.nlm.nih.gov/gene/7099 (accessed on 29 August 2021)). There are four transcripts for this gene (ensemble.org). This gene encodes the Toll-like receptor 4 protein, a member of the Toll-like receptor (TLR) family as well. Figure 2D shows the subcellular localization of TLR4. The predicted network of protein–protein interactions of TLR4 protein is shown in Figure 2E and its Gene Coexpression matrix (Figure 2F) shows coexpression with LY86, CD14, and LY96 with scores of 0.301, 0.264, and 0.176, respectively (https://string-db.org (accessed on 29 August 2021)). Rs4986790 is an SNP located at Chromosome 9, position: 117713024 (forward strand) with three alleles (A, G, and T). A is the ancestral allele and the minor allele frequency for G equals 0.06. This is a missense mutation that causes the replacement of amino acid Aspartic acid with amino acid Glycine at position 299.

#### 2.1.3. Predicting the Effect of SNPs on Protein Function

**Figure 3:**
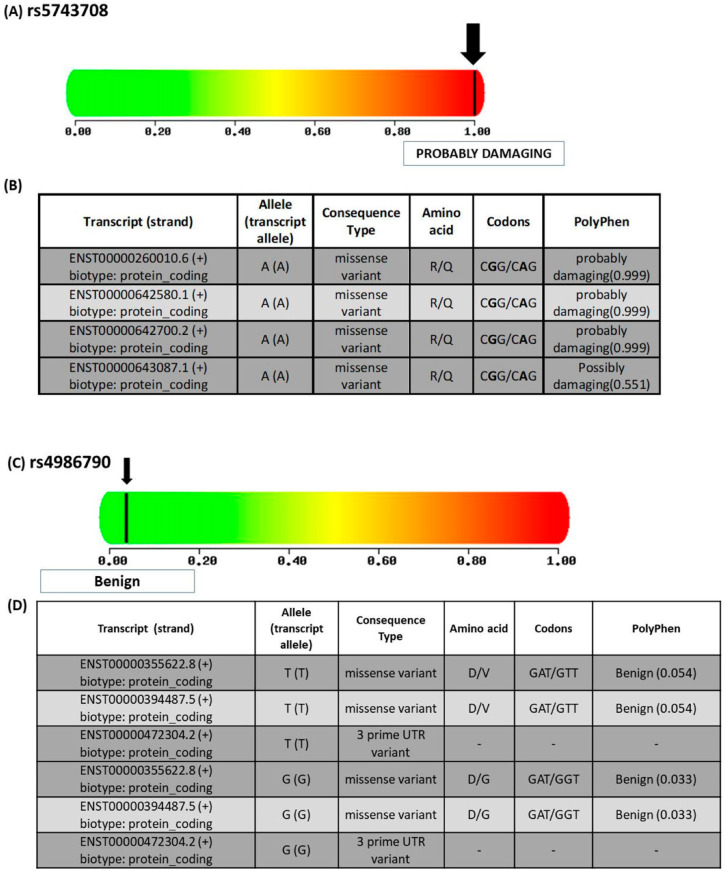
Functional and structural consequences of SNPs (**A**). Predicting the impact of rs5743708 on TLR2 function—the score ranged from benign (0) to damaging (1) (**B**). Table showing the transcripts of rs5743708, allele (transcript allele), consequence type, amino acid fate, codons, and PolyPhen score. R: Arginine, Q: Glutamine (ensemble.org). (**C**) Predicting the impact of rs4986790 on TLR4 function the score ranges from benign (0) to damaging (1) (**D**). Table showing the transcripts of rs4986790, allele (transcript allele), consequence type, amino acid fate, codons, and PolyPhen score. D: Aspartate, V: valine, G: Glycine (ensemble.org).

Five bioinformatics tools were used to predict the impact of rs4986790 and rs5743708 on the TLR4 and TLR2 proteins, respectively, to increase the accuracy of the results. For TLR4, all used bioinformatics tools predicted this variation to be neutral or benign as shown in Table 1. While for TLR2, all tools predicted this SNP to be damaging except SNPs and GO which predicted it to be neutral (Table 1). Figure 3 shows the structural and functional effects of SNPs.

#### 2.1.4. Identifying SNP Location on Protein Domains

Using InterPro revealed that rs5743708 was found to be located on the Toll/Interleukin-1 Receptor Homology (TIR) Domain (InterPro entry: IPR000157) in the TLR2 protein which is an essential domain for protein function, while rs4986790 location was found to belong to a superfamily called Leucine-rich repeat domain superfamily (InterPro entry: IPR032675).

#### 2.1.5. Prediction of Protein Stability with SNPs

I-Mutant 2.0 web server analyzed the effects of rs5743708 and rs4986790 SNPs on the stability of TLR2 and TLR4 proteins, respectively, by calculating free energy change values (DDG) and the Reliability Index value (RI). For TLR4, rs4986790 was found to decrease stability with RI = 3 and DDG = 0.38 Kcal/mol. While for TLR2, rs5743708 was found to decrease stability with RI = 8 and DDG = −0.71 Kcal/mol.

#### 2.1.6. Conservation Analysis

**Figure 4:**
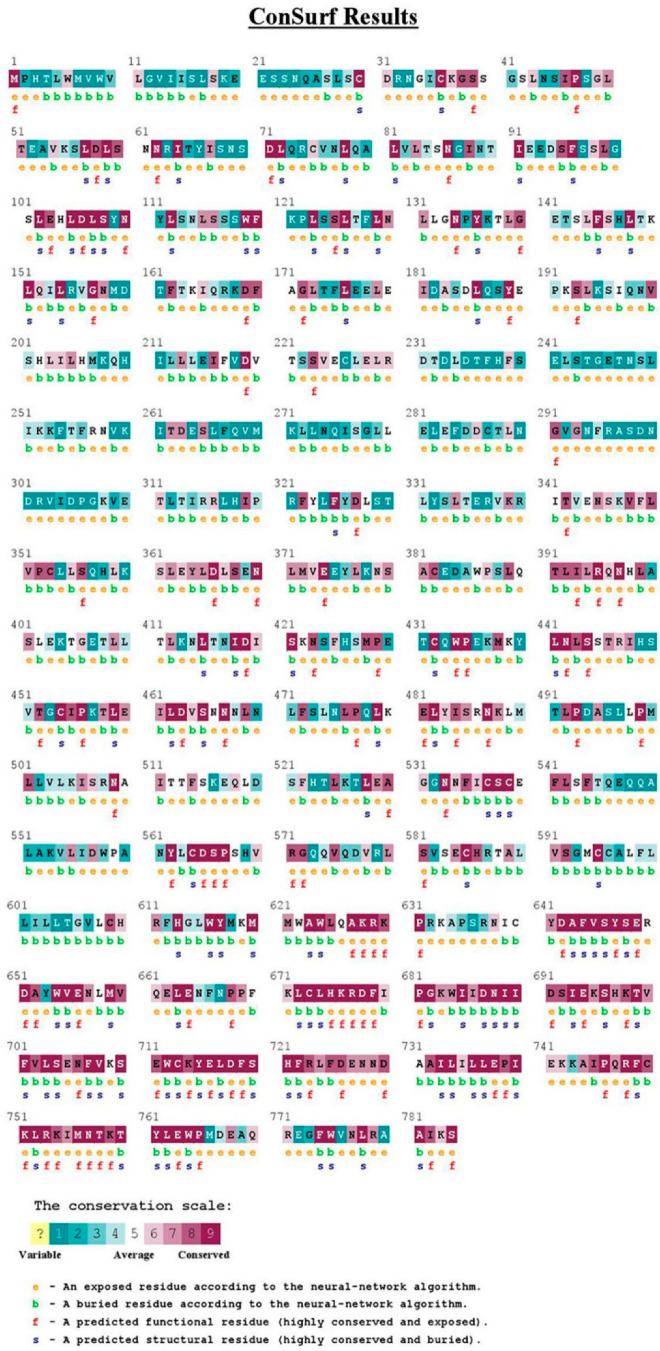
Evolutionary conservation analysis of TLR2 by Consurf.

**Figure 5:**
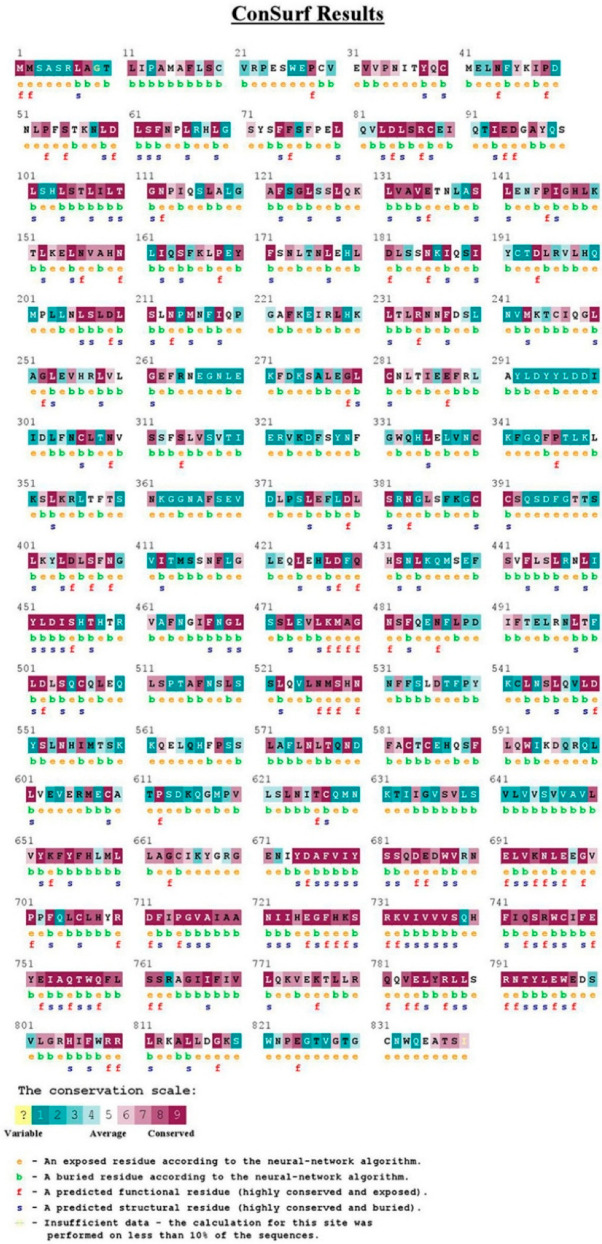
Evolutionary conservation analysis of TLR4 by Consurf.

TLR2 and TLR4 proteins were analyzed by the ConSurf server to perform an evolutionary conservation analysis of their amino acid positions (Figure 4 and Figure 5), respectively. In TLR2, position 753 (R753) was found to be an exposed and functional residue with high conservation. While in TLR4, position 299 (D299) was found to be an exposed and variable residue.

#### 2.1.7. Identifying the Structural Effects of SNPs

**Figure 6:**
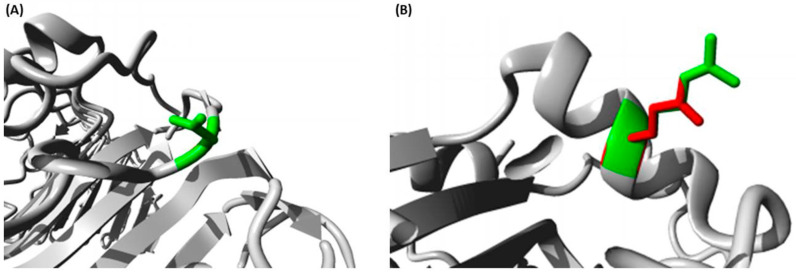
HOPE illustration of mutation structural impacts (**A**). Project HOPE illustration of the structural replacement of Aspartic acid with Glycine at position 299 in the TLR4 protein (colored with grey). The side chain of the wild type is colored in green while Glycine only has a hydrogen atom in its side chain (**B**). Project HOPE illustration of the structural replacement of Arginine with Glutamine at position 753 in the TLR2 protein (colored with grey). The side chain of the wild type is colored green while the side chain of the mutant type is colored red.

Using Project HOPE to analyze rs4986790 in TLR4, the new Glycine residue was found to differ in size and charge from the wild residue (Aspartic Acid) which could lead to a loss of interactions (Figure 6A). There was a difference in hydrophobicity too, which could cause loss of hydrogen bonds with possible disturbance of correct folding. Moreover, this replacement leads to an inability to form a Cysteine Bridge with its importance to protein stability, thus affecting the 3D structure of the protein and protein stability. In addition, Glycine flexibility affects the needed stability at that position. Meanwhile, analyzing rs5743708 in TLR2 revealed differences in size and charge between wild and mutant amino acids which could cause a loss of interactions (Figure 6B). Moreover, the different properties could lead to disturbance and elimination of (TIR) Domain function with its importance for protein function.

### 2.2. Demographic and Microbiological Data

A total of seventy-five Egyptian unrelated patients were included in the study. All participants had developed an infection. The patients were followed up to assess sepsis and septic shock, and the demographic features and the clinical characteristics of ICU-admitted patients according to developing sepsis are presented in Table 2. The two groups had significant differences in age factor, APACHE score at admission, and some categories of admissions. Causative organisms are listed (Table 2). There was no statistically significant difference between any of the causative organisms and developing sepsis.

### 2.3. Allele Frequencies of TLR2 and TLR4 Genes in the Study Population

Genotype and allele frequencies for TLR2 and TLR4 were detailed in Table 3. For TLR4, the frequency of wild-type genotype AA was 91%, while the heterozygous genotype AG was 8%, and the mutant genotype GG was 1%. The genotype frequencies followed the genotype frequencies expected by Hardy–Weinberg equilibrium (*p* > 0.05). For TLR2 the frequency of wild-type genotype GG was 92%, while the heterozygous genotype GA was 5%, and the mutant genotype AA was 3%. The genotype frequencies did not follow the genotype frequencies expected by Hardy–Weinberg equilibrium (*p* < 0.05).

Genotype association models for the risk of sepsis were analyzed and a significant association was found between TLR2 Arg753Gln SNP and sepsis under the over dominant model (*p* = 0.043), but in the TLR4 polymorphism this difference did not reach statistical significance (Table 4).

### 2.4. TLR2 and TLR4 Polymorphisms in Relation to Clinical and Laboratory Data

The association of single nucleotide polymorphisms (SNPs) with clinical and laboratory characteristics data is studied in Table 5. There was a statistically significant association between the *TLR4* polymorphism (rs4986790) and infection with *Acinetobacter baumannii* (*p* = 0.001) and infection with undetermined Gram (−) bacilli. Moreover, a statistically significant association was found between the *TLR4* polymorphism (rs4986790) and post-surgical patients’ admission category referred to ICU (*p* = 0.033). In addition, the *TLR4* polymorphism (rs4986790) had a significant association with the selection of Azithromycin as an empirical antibiotic (*p* = 0.003), and Imipenem antibiotic (*p* = 0.024), while the *TLR2* polymorphism (rs5743708) had an association with the selection of Teicoplanin (*p* < 0.001) and with Ampicillin + Sulbactam (*p* = 0.022). The selected empirical antibiotic depended on patient status and the severity of infection.

### 2.5. Multivariate Analysis in Relation to Developing Sepsis

A multivariate analysis was performed to determine which variable was independently associated with the risk of sepsis (Table 6). Only age was found to be independently associated with the risk of sepsis with a *p*-value of 0.009.

### 2.6. Survival Analysis

Survival analysis was performed with the usage of Log-rank, Breslow, and Tarone–Ware tests which showed significance only with the length of stay (0.001, 0.001, and 0.001), respectively, and with the post-surgical category of admission with a log-rank test (0.03), as shown in Table 7.

In addition, Cox regression analysis was applied to the data to determine if any of these variables were independently associated with the duration of survival (Table 8). Hazard risk for TLR2 was 1.89 and hazard risk for TLR4 was 2.25 but these results did not reach significance, so the effect of TLR gene status during time remained constant.

## 3. Discussion

The remarkable importance of TLR2 and TLR4 in our immune system and in modulating our response to infection suggested potential roles of their important variants, Arg753Gln and Asp299Gly, in increasing susceptibility to infection and sepsis as well.

Different bioinformatics approaches were utilized in our analysis. Investigating the impacts of our variants depended on five various tools with various approaches to achieve a high robustness and effectiveness. While rs4986790 was predicted to possess a benign impact on TLR4 by all tools, rs5743708 was predicted by all tools except SNPs and GO to possess a damaging impact on TLR2. Moreover, the SNPs’ positions on the domains of their proteins were determined by InterPro, revealing the presence of rs5743708 on the important TIR domain. The TIR domain has a crucial role in the activation of TLR pathways [26]. Therefore, it is anticipated that this mutation could affect its protein function. In addition, since protein function and structure are critically dependent on its stability [27], the impacts of rs5743708 and rs4986790 on their proteins’ stability were investigated revealing how proteins’ stability was reduced by these SNPs. Furthermore, concerning the relationship between high scores of conservation and functionally significant residues [28], the conservation analysis was intended to anticipate those SNPs which could affect the significant functions. Rs5743708 of *TLR2* was found to be a functional residue with high conservation. On the contrary, rs4986790 of *TLR4* was found to be a variable residue. In addition, both rs5743708 and rs4986790 were anticipated to induce structural impacts on TLR2 and TLR4, respectively using the HOPE bioinformatics server.

In our prospective study, the genotype frequencies for *TLR4* were in accordance with Hardy–Weinberg equilibrium. On the contrary, the genotype frequencies for *TLR2* were not in accordance with the Hardy–Weinberg equilibrium, and this aberrant result was also found by Saleh et al. in the Egyptian population, in his study about Toll-like receptor-2 polymorphisms and the susceptibility to pulmonary and peritoneal tuberculosis [29] which may require further investigation. The different prevalence of these SNPs between different populations have been steadily observed by different researchers [13,17,18] with obvious differences between Asian, African, and European ethnicities for both SNPs. These different distribution patterns between populations were suspected to be responsible for different susceptibility patterns to infectious diseases and other serious diseases such as coronary artery disease and type 2 Diabetes as well [18,30].

In our study, there was a significant association between TLR2 Arg753Gln polymorphism and sepsis under the over-dominant model (*p* = 0.043), while the TLR4 polymorphism did not show such significance. Some other investigators reached the same results in some populations despite the observed conflict between studies. A meta-analysis study conducted by Gao and colleagues found an association in this study between Arg753Gln SNP and the risk of sepsis among critically ill adult patients in Europe. Meanwhile, this study also shed light on the issue of the conflicting results regarding the TLR2 polymorphism and developing sepsis [21]. The TLR4 polymorphism studies also showed conflicting results; a study conducted in France by Lorenz et al. found that the Asp299Gly and Thr399Ile polymorphisms of TLR4 might potentially be linked to Gram-negative septic shock [19]. On the contrary, some studies showed an absence of association between TLR4 SNP and sepsis; a study conducted by Kumpf et al. found no association between Asp299Gly and Thr399Ile polymorphisms of TLR4 and the incidence of sepsis syndrome or the type of organisms causing surgical infection in German adults [20]. In addition, another study by Shan Xo et al. in Wenzhou found that the Asp299Gly and Thr399Ile polymorphisms may not correlate with susceptibility to sepsis in Chinese Han children [31]. These conflicting results can be seen frequently among different ethnic groups in these types of genetic association studies investigating diseases that depend on several genetic factors [32], as sepsis is believed to be initiated and augmented by multiple genes and there is no full control over sepsis by a single gene [33,34]. Consequently, the various frequency of different SNPs in different ethnic groups, and the difference in the penetration and the effect of SNPs because of other factors such as gender or age variations in different studies could explain these conflicting results among different populations.

Developing infection with *Acinetobacter baumannii* was found to have a statistically significant association with the TLR4 polymorphism (*p* = 0.001). This finding is in agreement with a recent study conducted by Chatzi et al. who found that the Asp299Gly and Thr399Ile polymorphisms of TLR4 could play an essential role in developing multidrug resistance to *Acinetobacter baumannii* in CNS infections [35]. In addition, other researchers have confirmed the role of TLR4 in *Acinetobacter baumannii* infection in vitro and in vivo and found that the production of IL-8 by epithelial A549 cells in the human lung as a response to *Acinetobacter baumannii* required both TLR2 and TLR4 [36]. However, other studies showed that the recognition of *Acinetobacter baumannii* depends on TLR4 rather than TLR2, as TLR4 is the dominant receptor in this type of recognition. Knapp et al. found that TLR4-deficient mice, not TLR2-deficient mice (with intranasal inoculation of *Acinetobacter baumannii* Lipopolysaccharides) showed the impaired production of TNFa in bronchi alveolar lavage fluid and the impaired recruitment of polymorph nuclear cells, compared with Wild Type mice [37]. Moreover, Kim et al. found that the production of *Acinetobacter baumannii*-induced cytokines was impaired with TLR4-deficient bone marrow-derived macrophages or dendritic cells, while it was not the case with TLR2-deficient macrophages [38]. Besides, Erridge et al. found that the activation of human monocytes (resulting from phenol water re-extracted Lipopolysaccharides from *Acinetobacter baumannii*) was the responsibility of the TLR4 signaling pathway [39]. This association between the TLR4 polymorphism and this virulent bacterium could allow proper management and prevention measures where high rates of *Acinetobacter baumannii* infection are found. This could be an important step towards the individualization of host susceptibilities towards virulent microorganisms in intensive care units.

Our study also found a significant association between the *TLR4* polymorphism (rs4986790) and the post-surgical category among patients referred to ICU. This role of the TLR4 polymorphism in post-surgery was investigated by a clinical study conducted by Koch and colleagues who found that the presence of a *TLR4* polymorphism influenced the immune–endocrine stress response which resulted from the systemic inflammation caused by major surgery. They found decreased serum concentrations of ACTH, IL-8, IL-10, and GM-CSF postoperatively in those surgical patients who carried that polymorphism [40]. This might explain this significant association found in our study.

The multivariate analysis was also performed to analyze the effects of our variables on the development of sepsis syndrome, but it was only the age factor that was found to have an independent association with the risk of sepsis in our study group. The age factor is a well-identified risk factor for developing this syndrome [41].

Survival analysis found that the length of stay and the surgical category of admission had a significant association with time of survival in intensive care units. Our results are in agreement with several studies that found an association between prolonged ICU stay and higher hospital mortality as well. Those patients, with an ICU length of stay of 14 days or longer, were found to have a mortality rate of more than 50% [42,43].

Overall, our study is characterized by the usage of both experimental and in silico methods. Our investigation showed promising results regarding the analysis of the role of TLRs variants in infection and sepsis. However, our study had its limitations as in most genetic polymorphism studies, as the number of patients carrying the variant alleles was relatively small due to the small number of these polymorphisms in the general population. As a result, there is a need for multi-center studies conducted on a larger scale to validate these findings.

## 4. Materials and Methods

### 4.1. Ethics Statement

The study protocol was approved by Scientific Research Ethics Commission at Suez Canal University (reference No. 201709MH1). All subjects or their next of kin gave informed consent before inclusion in the study.

### 4.2. In Silico Analysis

#### 4.2.1. General Information

National Center for Biotechnology Information (NCBI) and Ensembl databases were used to retrieve general information about *TLR2* and *TLR4* genes. Subcellular localization was retrieved from compartments.jensenlab.org mainly and genecards.org. Gene coexpression and predicted protein–protein interactions were obtained from the String Biological database. General information about rs5743708 and rs4986790 were brought from the dbSNP and Ensembl databases. (https://web.expasy.org (accessed on 29 August 2021)) was used for retrieving data about the variants’ effect on sequences of our proteins with these data gained from UniProtKB/Swiss-Prot databases.

#### 4.2.2. Predicting the Effect of SNPs on Protein Function

Five bioinformatics tools were used to predict the effect of SNPs on protein function to increase the strength and accuracy of results; 1-SIFT (Sorting Intolerant from Tolerant) (https://sift.bii.a-star.edu.sg/ (accessed on 30 August 2021)). SIFT depends on sequence homology in addition to the physical properties of amino acids to predict the effect of missense mutations on protein function [44]. 2-PolyPhen-2 (Polymorphism Phenotyping v2) (http://genetics.bwh.harvard.edu/pph2 (accessed on 30 August 2021)). PolyPhen-2 uses comparative and physical approaches to predict the effect of amino acid substitution [45]. 3-PANTHER (Protein Analysis Trough Evolutionary Relationship) (http://www.pantherdb.org/tools/csnpScoreForm.jsp (accessed on 30 August 2021)). This method depends on calculating the evolutionary preservation of an amino acid to predict the likelihood that a nonsynonymous SNP could cause a functional impact on the protein [46]. 4- PROVEAN (Protein Variation Effect Analyzer) (http://provean.jcvi.org/seq_submit.php (accessed on 30 August 2021)). PROVEAN uses blast hits to calculate the delta alignment score and computes the PROVEAN score finally with a cutoff at −2.5 [47]. 5-SNPs and GO (https://snps.biofold.org/snps-and-go/snps-and-go.html (accessed on 30 August 2021)). SNPs and GO depend on protein functional annotation to predict the impact of variations [48].

#### 4.2.3. The Identification of SNP Location on Protein Domains

The locations of SNPs on conserved domains on TLR2 and TLR4 proteins were identified using the InterPro bioinformatics tool (https://www.ebi.ac.uk/interpro/ (accessed on 30 August 2021)), a bioinformatics tool that could perform functional analysis of protein and identify domains and functional sites [49].

#### 4.2.4. The Prediction of Protein Stability with SNPs

We used I-Mutant 2.0 (https://folding.biofold.org/i-mutant/i-mutant2.0.html (accessed on 30 August 2021)) to predict the stability of the TLR2 and TLR4 proteins with rs5743708 and rs4986790 SNPs, respectively [50]. I-Mutant 2.0 is considered a support vector machine that was tested depending on the ProTherm database which contained the largest experimental data about stability changes with protein mutations [51].

#### 4.2.5. The Identification of Evolutionarily Conserved Positions in a Protein Sequence

This identification was performed using the ConSurf server (https://consurf.tau.ac.il (accessed on 30 August 2021)) which depends on phylogenetic relations between homologous sequences to identify the evolutionary conservation of amino acids in protein sequences [28,52].

#### 4.2.6. The Identification of Structural Effects of SNPs

Structural effects of rs5743708 and rs4986790 SNPs on TLR2 and TLR4, respectively, were analyzed using HOPE (https://www3.cmbi.umcn.nl/hope/ (accessed on 30 August 2021)) which is a mutant analysis server that could analyze the effects of SNPs on protein structure [53].

### 4.3. The Study Design

This was a prospective observational study that was conducted in intensive care units in Suez Canal University Hospitals, Ismailia, Egypt, for seven months. All ICU Patients who contracted infections with a positive culture or a chest X-ray were included in the study group. All included patients were Egyptian adults of both sexes. Exclusion criteria were patients younger than 18 years old, pregnancy, immune suppression, and patients with radiation therapy or chemotherapy.

Once admitted, general examination and clinical status were assessed for patients; both Acute Physiology and Chronic Health Evaluation (APACHE II) scores and sequential organ failure assessment (SOFA) scores were measured. In addition to vital signs check (blood pressure, heart rate, respiratory rate, central venous pressure, and temperature) and laboratory analyses such as complete blood count, blood sugar, CRP, blood urea nitrogen, serum calcium, potassium, sodium, aspartate aminotransferase, alanine aminotransferase, and arterial blood gas analysis were carried out.

The patients were further followed up to assess infection, sepsis, and septic shock. Routine cultures of sputum, blood, urine, and pus were collected to determine the presence of infection and identify the causing organism. Assessment of sepsis and septic shock was performed by daily evaluation for sepsis or septic shock. Sepsis and septic shock were defined and diagnosed according to “The Third International Consensus Definitions for Sepsis and Septic Shock (Sepsis-3)” [4].

### 4.4. Samples Collection

Two milliliters of venous blood sample were collected into EDTA tubes from all admitted patients in the study group under complete aseptic conditions and stored at −80 °C until processed for DNA extraction.

### 4.5. Genotyping

Genomic DNA was extracted from venous blood with a QIAamp DNA Blood Mini kit (Cat. No. 51104; QIAGEN, Hilden, Germany) according to the manufacturer’s protocol. The measurement of both the concentration and purity of the extracted DNA was performed by NanoDrop ND-1000 (NanoDrop Tech., Inc., Wilmington, DE, USA).

Genotyping for the *TLR4* gene polymorphism (Asp299Gly; rs4986790) and *TLR2* gene polymorphism (Arg753Gln; rs5743708) was performed using real-time polymerase chain reaction technology using TaqMan allelic discrimination assay. The required reagents for the TaqMan assay including TaqMan genotyping assay and TaqMan genotyping master mix were brought from Applied Biosystems (Foster City, CA, USA). The assay ID for rs5743708 is C_27860663_10 and for rs4986790 is C_11722238_20. PCR was run with a total reaction volume of 25 μL reaction volume. The components of PCR reaction were 12.5 μL TaqMan genotyping master mix; No AmpErase UNG (2×), genomic DNA (20 ng) diluted to 11.25 μL with DNase-RNase free water, and 1.25 μL TaqMan SNP genotyping assay mix (Cat. No. 4351379, Applied Biosystems, Foster City, USA). Nuclease-free water was used as a negative control.

The PCR amplification was carried out in a StepOne™ real-time PCR system (Applied Biosystems, Foster City, CA, USA) according to the following conditions: a hold cycle (95 °C for 10 min) followed by a 40-cycle PCR consisting of 95 °C for 15 s and 60 °C for one minute. SDS software version 1.3.1 (Applied Biosystems) was used for allelic discrimination. Genotyping was performed with blindness to sepsis/non-sepsis status.

### 4.6. Statistical Analysis

Statistical analysis was carried out using Microsoft^®^ Excel 2010 and the “Statistical Package for the Social Sciences (SPSS) for windows” software, version 24. Odds ratios (OR) with a 95% confidence interval (CI) were calculated. Descriptive statistics were expressed as percentages for qualitative variables and mean ± standard deviation (SD) for quantitative variables. Testing differences between septic patients and no septic patients were performed using Student’s *t*-test, Chi-square (χ^2^) test, or Fisher’s exact tests. *p*-value was considered statistically significant below 0.05. The Hardy–Weinberg equilibrium (HWE) was calculated by the Online Encyclopedia for Genetic Epidemiology (OEGE) software (http://www.oege.org/software/hwe-mrcalc.shtml (accessed on 10 March 2019)). The relationship between the risk factors including our polymorphisms and the development of sepsis was further determined using logistic regression after adjustment of factors. Survival analysis was performed as well. Log-rank, Breslow, and Tarone–Ware tests were used to find Kaplan–Meier estimates for survival. Cox regression analysis was applied to the data to determine if any of the variables were independently associated with the duration of survival.

## 5. Conclusions

Rs5743708 was predicted by nearly all used bioinformatics tools to possess a damaging impact on TLR2 which was not the case with rs4986790 of TLR4. Meanwhile, the conducted pilot study concluded that the *TLR2* genotype may be a risk factor for sepsis in adult patients, Moreover, our study showed that Asp299Gly polymorphism in TLR4 may be associated with an increased risk of *Acinetobacter baumannii* infection. In addition, a significant association was found between the *TLR4* polymorphism and the post-surgical category of patients admitted to intensive care units. Identification of the role of *TLR2* and *TLR4* polymorphisms in developing infection and sepsis could allow early prediction, prevention, and management of these serious diseases.

## Figures and Tables

**Table 1:** Predicting the effect of SNPs on protein function using bioinformatics tools.

SNP	Amino Acid Change	SIFT	Polyphen2	PANTHER	PROVEAN	SNPs and GO
rs4986790	D299G	Tolerated	Benign	probably benign	Neutral	Neutral
rs5743708	R753Q	Deleterious	Probably Damaging	probably damaging	Deleterious	Neutral

**Table 2:** Demographic and clinical features of ICU admitted patients with and without sepsis.

Variables		All	No Sepsis	Sepsis	*p*-Value	OR (95% CI)
Demographic Characteristics						
Number		75 (100%)	48 (64.0%)	27 (36.0%)		
Age, years	Mean ± SD	60.0 ± 17.6	55.5 ± 18.9	68.0 ± 11.5	0.003	
	≤40 years	14 (18.7%)	14 (29.2%)	0 (0.0%)	0.002	Reference
	≤60 years	20 (26.7%)	14 (29.2%)	6 (22.2%)		13.0 (0.67–252.6)
	>60 years	41 (54.7%)	20 (41.6%)	21 (77.8%)		30.41 (1.7–543.6)
Sex	Male	47 (63.0%)	31 (64.6%)	16 (59.3%)	0.64	Reference
	Female	28 (37.0%)	17 (35.4%)	11 (40.7%)		1.25 (0.48–3.30)
Vital signs	HR	100.6 ± 21.3	101.2 ± 20.8	99.5 ± 22.4	0.75	
	MAP	81.7 ± 25	85.4 ± 27.4	75.2 ± 18.7	0.09	
Concomitant diseases						
Diabetes	Positive	29 (38.7)	17 (35.4%)	12 (44.4%)	0.44	1.46 (0.56–3.82)
Hypertension	Positive	42 (56.0%)	26 (54.2%)	16 (59.3%)	0.67	1.23 (0.47–3.20)
Vascular disease	Positive	27 (36.0%)	15 (31.3%)	12 (44.4%)	0.25	1.76 (0.66–4.66)
Chronic lung disease	Positive	6 (8.0%)	5 (10.4%)	1 (3.7%)	0.41	0.33 (0.04–2.99)
Chronic liver disease	Positive	7 (9.3%)	2 (4.2%)	5 (18.5%)	0.09	5.23 (0.94–29.10)
Chronic renal disease	Positive	17 (22.7%)	10 (20.8%)	7 (25.9%)	0.61	1.33 (0.44–4.02)
ICU assessment						
APACHE score	Mean ± SD	17.4 ± 8.3	15.8 ± 6.0	20.3 ± 10.8	0.024	
Glasgow scale	Mean ± SD	9.8 ± 4.4	9.4 ± 4.1	10.5 ± 4.8	0.29	
Length of stay, days	Mean ± SD	19.7 ± 15.9	17.6 ± 11.8	23.4 ± 21.1	0.13	
Consequence	Discharge	27 (36.0%)	19 (39.58%)	8 (29.63%)	0.69	Reference
	Transferred	5 (6.7%)	3 (6.25%)	2 (7.40%)		1.58 (0.22–11.3)
	Death	43 (57.3%)	26 (54.17%)	17 (62.96%)		1.55 (0.56–4.34)
OS, days	Mean ± SD	19.6 ± 17.2	17.5 ± 13.4	22.8 ± 22	0.33	
Admission category						
Renal	Positive	2 (2.7%)	1 (2.1%)	1 (3.7%)	0.67	1.81 (0.11–30.1)
Cardiovascular	Positive	3 (4%)	2 (4.2%)	1 (3.7%)	0.92	0.88 (0.08–10.2)
Infection	Positive	21 (28%)	7 (14.6%)	14 (51.8%)	0.001	7.23 (2.42–21.6)
Neurology	Positive	20 (26.7%)	17 (35.4%)	3 (11.1%)	0.022	0.27 (0.07–1.03)
Post-surgical	Positive	11 (14.7%)	6 (12.5%)	5 (18.5%)	0.47	1.59 (0.44–5.80)
Respiratory	Positive	10 (13.3%)	10 (20.8%)	0 (0.0%)	0.011	0.07 (0.00–1.19)
Trauma	Positive	3 (4%)	3 (6.3%)	0 (0.0%)	0.54	0.24 (0.01–4.75)
Other causes	Positive	5 (6.7%)	2 (4.2%)	3 (11.1%)	0.24	2.88 (0.45–18.4)
Variables		All	No sepsis	Sepsis	*p*-value	OR (95% CI)
Causative organism in culture						
*Enterobacter* spp.	Positive	6 (6.3%)	3 (6.3%)	3 (11.1%)	0.45	1.88 (0.35–10.01)
*Acinetobacter baumannii*	Positive	11 (11.5%)	7 (14.6%)	4 (14.8%)	0.97	1.02 (0.27–3.85)
*Candida albicans*	Positive	3 (3.1%)	1 (2.1%)	2 (7.4%)	0.25	3.76 (0.32–43.53)
*Escherichia coli*	Positive	15 (15.8%)	11(22.9%)	4 (14.8%)	0.40	0.59 (0.17–2.06)
*Gram negative bacilli*	Positive	3 (3.1%)	2 (4.2%)	1 (3.7%)	0.92	0.90 (0.08–10.45)
*Klebsiella pneumoniae*	Positive	20 (21.1%)	13 (27.1%)	7 (25.9%)	0.91	0.94 (0.32–2.75)
*Pseudomonas aeruginosa*	Positive	12 (12.6%)	6 (12.5%)	6 (22.2%)	0.27	2.00 (0.57–6.96)
*Staph* spp.	Positive	17 (17.9%)	12 (25.0%)	5(18.5%)	0.52	0.68 (0.21–2.20)
*Streptococcus* spp.	Positive	4 (4.2%)	3 (6.25%)	1 (3.7%)	0.63	0.58 (0.06–5.84)
*Aeromonas hydephila*	Positive	1 (1.1%)	1 (2.1%)	0 (0%)	1.00	0.58 (0.02–14.6)
*Proteus* spp.	Positive	1 (1.1%)	1 (2.1%)	0 (0%)	1.00	0.58 (0.02–14.6)
*Citrobacter* spp.	Positive	1 (1.1%)	1 (2.1%)	0 (0%)	1.00	0.58 (0.02–14.6)
*Serratia* spp.	Positive	1 (1.1%)	1(2.1%)	0 (0%)	1.00	0.58 (0.02–14.6)

Data are shown as a number (percentage) or number ± standard deviation. HR: heart rate in beats per minute; MAP: mean arterial pressure in mmHg, OS: Overall survival. Chi-square (χ^2^) or Fisher’s exact tests were used for qualitative variables and student’s *t*-test was used for quantitative attributes. OR (95% CI), odds ratio, and confidence interval. Statistical analysis at *p*-value < 0.05.

**Table 3:** Genotype and allele frequencies of *TLR2* and *TLR4* genes in the study population according to developing or not developing sepsis.

Variables	*TLR2* (rs5743708)				*TLR4* (rs4986790)			
	All	Non-Septic	Septic	*p*-Value	All	Non-Septic	Septic	*p*-Value
Genotype frequencies								
A/A	2 (3)	1 (2)	1 (4)	0.22	68 (91)	45 (94)	23 (85)	0.20
G/A	4 (5)	1 (2)	3 (11)		6 (8)	2 (4)	4 (15)	
G/G	69 (92)	46 (96)	23 (85)		1 (1)	1 (2)	0 (0)	
Allele frequencies								
A	8 (5)	3 (3)	5 (9)	0.10	142 (95)	92 (96)	50 (93)	0.39
G	142 (95)	93 (97)	49 (91)		8 (5)	4 (4)	4 (7)	
P _HWE_	0.009	0.032	0.180		0.180	0.063	1.00	

Data are shown as a number (percentage). Fisher’s Exact tests were performed. Statistical analysis at *p* value < 0.05.

**Table 4:** Genotype association models for sepsis risk assessment.

Model	Genotype	Non-Septic	Septic	Adjusted OR (95% CI) ^a^	*p*-Value
TLR2					
Codominant ^b^	G/G	46 (95.8%)	23 (85.2%)	Reference	
	A/G	1 (2.1%)	3 (11.1%)	11.42 (0.84–155.32)	0.12
	A/A	1 (2.1%)	1 (3.7%)	1.65 (0.09–29.49)	
Dominant	G/G	46 (95.8%)	23 (85.2%)	Reference	0.07
	A/G-A/A	2 (4.2%)	4 (14.8%)	5.34 (0.77–36.96)	
Recessive	G/G-A/G	47 (97.9%)	26 (96.3%)	Reference	0.79
	A/A	1 (2.1%)	1 (3.7%)	1.48 (0.09–25.46)	
Over-dominant	G/G-A/A	47 (97.9%)	24 (88.9%)	Reference	0.043
	A/G	1 (2.1%)	3 (11.1%)	11.27 (0.83–152.94)	
Log-additive	---	---	---	2.42 (0.61–9.56)	0.18
TLR4					
Codominant ^b^	A/A	45 (93.8%)	23 (85.2%)	Reference	0.11
	A/G	2 (4.2%)	4 (14.8%)	7.23 (0.77–67.86)	
	G/G	1 (2.1%)	0 (0%)	0.00 (0.00-NA)	
Dominant	A/A	45 (93.8%)	23 (85.2%)	Reference	0.16
	A/G-G/G	3 (6.2%)	4 (14.8%)	3.68 (0.57–23.57)	
Recessive	A/A-A/G	47 (97.9%)	27 (100%)	Reference	0.36
	G/G	1 (2.1%)	0 (0%)	0.00 (0.00-NA)	
Over-dominant	A/A-G/G	46 (95.8%)	23 (85.2%)	Reference	0.06
	A/G	2 (4.2%)	4 (14.8%)	7.49 (0.79–71.02)	
Log-additive	---	---	---	1.90 (0.45–8.04)	0.37

Values are shown as numbers (%). Chi-square (χ^2^) or Fisher’s exact tests were used. OR (95% CI), odds ratio, and confidence interval. ^a^ adjusted for confounding factors (age and sex). ^b^ represented both heterozygote and homozygote comparison models.

**Table 5:** Analysis for the association of variants with clinical and laboratory characteristics.

**Variables**		***TLR2* (rs5743708)**	***TLR4* (rs4986790)**
		***p*-Value**	***p*-Value**
Demographic	Age, years	0.84	0.99
	Sex	0.27	0.71
Vital signs	HR, beats/min	0.27	0.47
	MAP, mm Hg	0.70	0.84
	SBP, mm Hg	0.70	0.86
	DBP, mm Hg	0.80	0.92
Concomitant diseases	Diabetes	0.80	0.08
	Hypertension	0.95	0.06
	Vascular dis	0.14	0.43
	Chronic lung disease	0.75	0.71
	Chronic liver disease	0.49	0.67
	Chronic renal disease	0.31	0.80
ICU assessment	APACHE score	0.75	0.70
	Glasgow scale	0.89	0.24
	Length of stay	0.84	0.36
	Sepsis	0.22	0.20
	Septic shock	0.74	0.44
	Death	0.75	0.46
	Overall survival	0.52	0.06
Admission category (cause of admission)	Renal	0.91	0.90
	Cardiovascular	0.87	0.25
	Infection	0.07	0.38
	Neurology	0.30	0.77
	Post-surgical	0.70	0.033
	Respiratory	0.22	0.55
	Trauma	0.87	0.85
	Other causes	0.79	0.75
Biochemical data	WBC, ×10^3^ cells/μL	0.56	0.16
	HB, g%	0.08	0.31
	Creatinine, mg/dL	0.98	0.24
Causative organism	*Enterobacter* spp.	0.07	0.05
	*Acinetobacter* spp.	0.70	0.001
	*Candida* spp.	0.08	0.85
	*E. coli*	0.44	0.38
	*Gram (−) bacilli*	0.87	<0.001
	*Klebsiella* spp.	0.30	0.69
	*Pseudomonas* spp.	0.73	0.90
	*Staph* spp.	0.63	0.80
	*Streptococcus* spp.	0.83	0.80
	*Aeromonas* spp.	0.95	0.94
	*Proteus* spp.	0.95	0.94
	*Citrobacter* spp.	0.95	0.94
	*Serratia* spp.	0.95	0.94
**Variables**		***TLR2* (rs5743708)**	***TLR4* (rs4986790)**
		***p*** **-Value**	***p*** **-Value**
Type of culture	Blood	0.68	0.76
	Sputum	0.77	0.29
	Urine	0.47	0.41
	Pus	0.35	0.73
	CSF	0.95	0.94
No of infections		0.48	0.94
Empirical antibiotic	No of antibiotics	0.05	0.77
	Cefoperazone	0.91	0.90
	Ceftazidime	0.57	0.90
	Levofloxacin	0.47	0.14
	Cefepime	0.16	0.23
	Ampicillin + sulbactam	0.022	0.70
	Imipenem	0.22	0.024
	Meropenem	0.95	0.94
	Ertapenem	0.75	0.69
	Azithromycin	0.95	0.003
	Rifampicin	0.95	0.94
	Teicoplanin	<0.001	0.94
	Cefotaxime	0.57	0.90
	Piperacillin	0.95	0.94

Chi-square (χ^2^) or Fisher’s exact tests were used for qualitative variables and student’s *t*-test was used for quantitative attributes. Statistical analysis at *p* value < 0.05.

**Table 6:** Multivariate analysis for the risk of sepsis in ICU-admitted patients.

Risk Factors	OR	95% CI (Lower)	95% CI (Upper)	*p*-Value
Age	0.940	0.897	0.984	0.009
Sex (female)	0.473	0.116	1.925	0.30
HR, beats/min	1.003	0.974	1.033	0.83
MAP, mm Hg	0.898	0.654	1.233	0.51
SBP, mm Hg	1.022	0.915	1.142	0.70
DBP, mm Hg	1.123	0.901	1.400	0.30
WBC, ×10^3^ cells/μL	0.926	0.854	1.003	0.06
HB, g%	0.875	0.666	1.150	0.34
Creatinine, mg/dL	0.938	0.724	1.217	0.63
APACHE score	0.942	0.815	1.088	0.42
Glasgow scale	0.962	0.800	1.156	0.68
Length of stay	0.962	0.917	1.008	0.11
TLR2 (A/G)	0.082	0.001	6.474	0.26
TLR2 (G/G)	1.939	0.084	44.584	0.68
TLR4 (A/G)	0.090	0.005	1.785	0.11
TLR4 (G/G)	NA	NA	NA	1.00

OR: odds ratio; CI: confidence interval. Binary logistic regression analysis was performed.

**Table 7:** Survival analysis in ICU-admitted patients.

Variables		Overall Comparisons		
		Log Rank	Breslow	Tarone–Ware
Demographic data	Age	0.44	0.36	0.35
	Sex	0.23	0.50	0.36
Vital signs	HR	0.61	0.99	0.84
	MAP	0.86	0.69	0.75
	SBP	0.45	0.46	0.44
	DBP	0.63	0.64	0.64
Concomitant disease	Diabetes	0.87	0.62	0.85
	Hypertension	0.12	0.26	0.17
	Vascular disease	0.39	0.28	0.28
	Chronic liver disease	0.58	0.50	0.50
	Chronic renal disease	0.42	0.55	0.48
ICU assessment	APACHE score	0.81	0.84	0.76
	Glasgow scale	0.51	0.54	0.57
	Length of stay	<0.001	<0.001	<0.001
	Sepsis	0.91	0.82	0.78
	Septic shock	0.94	0.69	0.74
	No empirical drug	0.06	0.09	0.06
Admission category	Renal	0.53	0.54	0.54
	Cardiovascular	0.30	0.56	0.44
	Infection	0.79	0.86	0.82
	Neurology	0.33	0.29	0.31
	Post-surgical	0.030	0.09	0.06
	Respiratory	0.61	0.74	0.62
	Trauma	0.32	0.39	0.37
	Other causes	0.26	0.59	0.40
Lab data	WBC, ×10^3^ cells/μL	0.66	0.93	0.84
	HB, g%	0.51	0.69	0.61
	Creatinine, mg/dL	0.71	0.42	0.55
	No of infection	0.48	0.47	0.54
Molecular analysis	TLR2	0.38	0.25	0.27
	TLR4	0.63	0.39	0.43
	Combined	0.12	0.35	0.22

Survival time is shown as mean and standard error, HR: Hazard ratio, CI; confidence interval. Log-rank, Breslow, and Tarone–Ware tests were used to find Kaplan–Meier estimates for survival. Quantitative variables were categorized by their medians.

**Table 8:** Multivariate analysis for the risk of mortality in ICU-admitted patients.

Variables		HR	95% CI	*p*-Value
Demographic data	Age	1.90	(0.47–7.57)	0.36
	Sex	0.42	(0.09–1.93)	0.27
ICU assessment	APACHE score	1.41	(0.31–6.24)	0.65
	Glasgow scale	1.86	(0.38–8.89)	0.44
	Septic shock	0.55	(0.10–2.90)	0.48
	No empirical drug	0.32	(0.04–2.06)	0.62
	No of infection	1.76	(0.19–16.23)	0.23
Molecular analysis	TLR2	1.89	(0.08–43.58)	0.69
	TLR4	2.25	(0.48–10.43)	0.30

HR: hazard risk, CI: confidence interval. Cox Proportional Hazard Regression analysis was performed.

## Data Availability

All supporting data of the study are available from the corresponding authors upon request.
